# Case report: A proposed role for cardiopulmonary exercise testing in detecting cardiac dysfunction in asymptomatic at-risk adolescents

**DOI:** 10.3389/fped.2023.1103094

**Published:** 2023-04-06

**Authors:** Timothy Edwards, Emir Tas, Kenneth Leclerc, Elisabet Børsheim

**Affiliations:** ^1^ Arkansas Children's Nutrition Center, Little Rock, AR, United States; ^2^ Arkansas Children's Research Institute, Little Rock, AR, United States; ^3^ Department of Pediatrics, University of Arkansas for Medical Sciences, Little Rock, AR, United States; ^4^ Department of Cardiology, Legacy Medical Group, Tualatin, OR, United States; ^5^ Department of Geriatrics, University of Arkansas for Medical Sciences, Little Rock, AR, United States

**Keywords:** case report, cardiac dysfunction, cardiopulmonary exercise testing (CPET), adolescents, family history

## Abstract

Noninvasive cardiopulmonary exercise testing (CPET) provides the valuable capacity to analyze pulmonary gas exchange and cardiovascular responses that can be used to differentiate normal cardiopulmonary responses from abnormal. This case report highlights a proposed role for CPET in identifying potential cardiac pathologies in at-risk adolescents. An abnormal CPET response in an asymptomatic adolescent revealed a family history of early-age CAD. The significance of the abnormal CPET response was further supported by the presence of an elevated concentration of circulating high sensitivity C-reactive protein (hs-CRP). These findings emphasize the importance of a thorough clinical evaluation in at-risk adolescents, as CPET can aid in the early detection and management of cardiac pathologies, especially when combined with other relevant biomarkers such as plasma hs-CRP concentration, which can further suggest underlying pathology. Management considerations using serial CPET evaluations are recommended. Thus, CPET abnormalities combined with elevated hs-CRP should be taken seriously and provide justification for further evaluation and monitoring in adolescents at risk for cardiovascular disease.

## Introduction

Cardiopulmonary exercise testing (CPET) is a well-recognized, but underutilized diagnostic and prognostic test for clinical evaluation in the symptomatic pediatric and adult populations. However, an effective preventive healthcare clinical workflow for apparently healthy asymptomatic individuals at-risk for future cardiovascular disease (e.g., family history, overweight/obesity, elevated hs-CRP) is not well established, especially in adolescents. As a non-invasive and radiation-free test, CPET provides a simultaneous evaluation of cardiovascular, pulmonary, and skeletal muscle responses that may detect early-stage disease states. Therefore, a clinical workflow utilizing CPET has the potential to improve the current preventive healthcare strategy.

The value of CPET is based on its ability to accurately measure oxygen consumption (V̇O_2_), which is the product of cardiac output and arterial-venous O_2_ difference. Cardiac output is measured indirectly by tracking HR and O_2_ pulse (surrogate of stroke volume) throughout incremental exercise. In healthy individuals, V̇O_2_ and HR increase linearly along with a linear or curvilinear O_2_ pulse when a linear ramping work rate (WR) protocol is used on a cycle ergometer. Key CPET parameters (ΔV̇O_2_/ΔWR, ΔHR/ΔWR, ΔO_2_ pulse/ΔWR) reflect how V̇O_2_, HR, and O_2_ pulse respond to increasing workloads and provide important insight into cardiovascular function (1, 2). Deviation of these parameters from their expected trajectories signals that a closer analysis may be warranted to assess any underlying pathology. Some disease states may not be detected using resting studies, as in the example of this case. Therefore, CPET may reveal abnormalities in asymptomatic adolescents that suggest possible underlying cardiopulmonary disease states. This case report describes the abnormal CPET response in an asymptomatic adolescent male that later revealed a significant family history risk factor of early-age CAD. The abnormal CPET response was further supported by the presence of an elevated concentration of circulating high sensitivity C-reactive protein (hs-CRP) which adds to the probability of an underlying cardiac pathology and emphasizes the importance of the need for a more thorough clinical evaluation.

## Case report

This paper reports on a 14-year-old Caucasian male participant with an overweight BMI percentile for age and sex who underwent a cardiopulmonary fitness test during a research study visit for apparently healthy adolescents. This Case Report comes from a follow-up study. The original study was approved by the Institutional Review Board at the University of Arkansas for Medical Sciences (IRB #206291), and the usage of data for this Case Report was approved by the same IRB (#263026).

The participant was enrolled in the original cohort study and was later recalled for a study visit when he was 14 years old, at which point the visit also included a CPET test. The case participant had no known previous or current medical history, was not taking any prescription medications, and was reportedly asymptomatic at the time of testing. Table 1 shows the demographics of both the case participant and a healthy control from the same study matched on age, sex and race, but with no significant family health history.

**Table 1:** Case participant and healthy control characteristics.

Demographic	Case participant	Control
Age (years)	14	14
Race	Caucasian	Caucasian
Sex	Male	Male
BMI percentile	87th	49th
Significant family history risk factors	Paternal early-age CAD	None
**Vitals**		
Resting heart rate (beats/min)	67	68
Resting blood pressure (mmHg)	115/63	125/80
**Blood measures**		
Hs-CRP (mg/L) (5%–95% interval in children: 0.1–2.8 mg/L) (5)	5.3	0.1
Glucose (mg/dl)	89	92
Total cholesterol (mg/dl)	109	133
HDL (mg/dl)	45	56
LDL (mg/dl)	52	76
Triglycerides (mg/dl)	74	41

BMI, body mass index; CAD, coronary artery disease; HDL, high density lipoprotein; LDL, low density lipoprotein. To convert total cholesterol, HDL cholesterol, and LDL cholesterol values to mmol/L, multiply by 0.0259; to convert triglyceride values to mmol/L, multiply by 0.0113.

**Figure 1:**
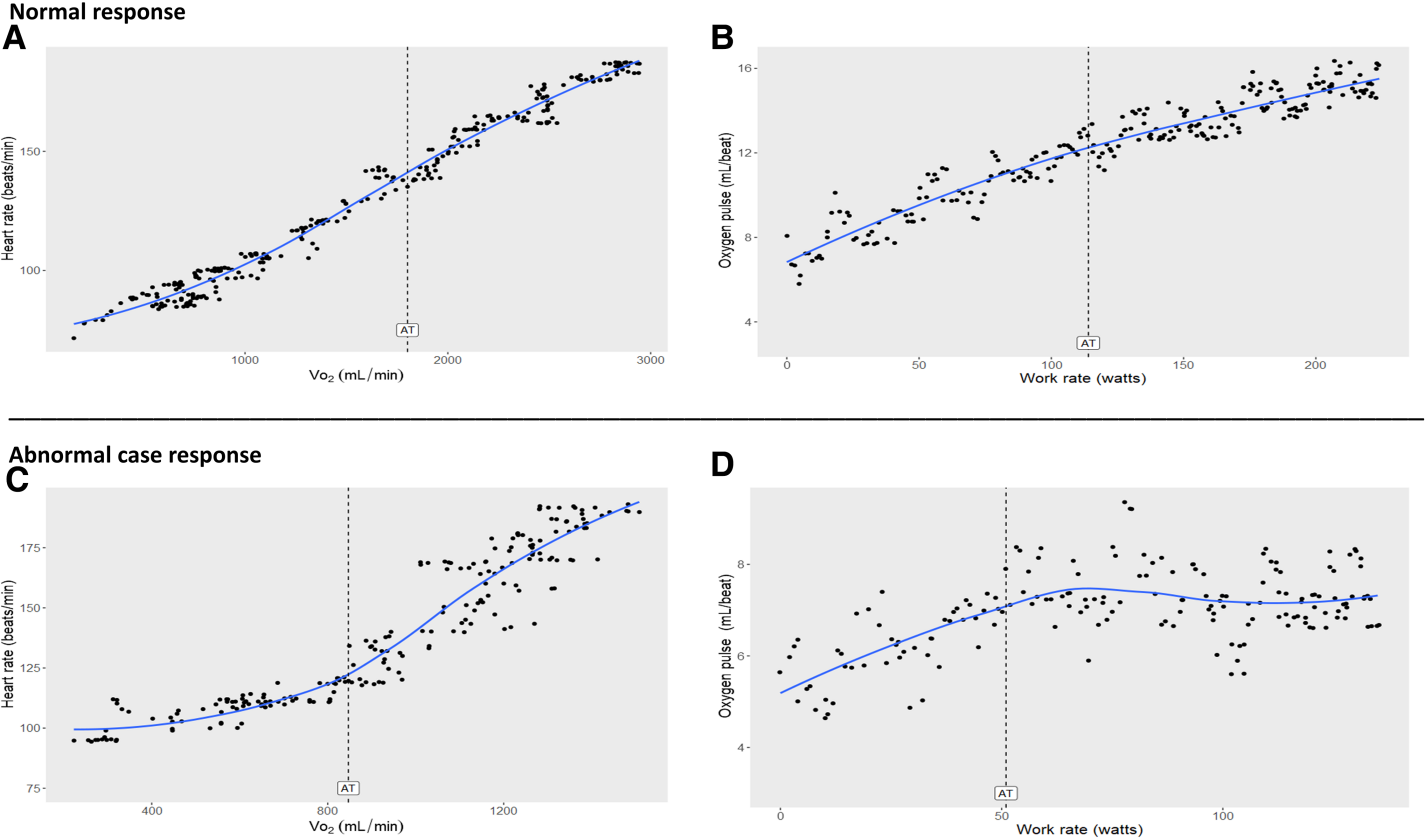
(**A,B)**: Normal cardiopulmonary exercise test (CPET) responses (normal cardiac function) in a healthy 14-year old male control. Note there is little change in trajectories after AT compared to before AT. (**C,D**): Abnormal CPET responses (cardiac dysfunction) from the case participant 14-year old male with elevated hs-CRP and a family history risk factor for early-age coronary artery disease. Note the significant steepening of heart rate trajectory with abrupt flattening of oxygen pulse after AT compared to before AT. AT, anaerobic threshold; Oxygen pulse, V̇O_2_/HR (index of stroke volume x arteriovenous oxygen difference); V̇O_2_, oxygen uptake. SI conversion factors: To convert V̇O_2_ and AT values to L/min, multiply by.001; to convert V̇O_2_/HR (oxygen pulse, ml/beat) to L/beat, multiply by.001.

Per the study protocol, the participant underwent fasting blood sampling to determine circulating levels of cardiometabolic and inflammatory markers. Vitals and blood sampling were followed by cardiopulmonary fitness testing *via* a maximum effort CPET using a metabolic cart (MGC Diagnostics Ultima PFX, St. Paul, Minnesota, USA) and cycle ergometer (Lode Corival, Gronigen, The Netherlands) with a linear ramp protocol of 20 watts per minute. The participant stopped the test due to leg fatigue and reached a respiratory exchange ratio of 1.16 (significant metabolic acidosis representing good effort), a peak HR of 93% of his age-predicted value (220-age), an anaerobic threshold (AT) of <40% of the predicted peak V̇O_2_, an O_2_ pulse at peak exercise (surrogate of peak stroke volume) of 58% of predicted, a ΔV̇O_2_/ΔWR of 6.4 ml·min^−1^·Watt^−1^ (64% of predicted) (3), and a peak V̇O_2_ of 21.3 ml·kg^−1^·min^−1^ (55% of predicted) (4). Blood pressure and electrocardiogram (EKG) were not measured during the exercise as peak V̇O_2_ was the study test's primary outcome. Graphically, his HR response as a function of V̇O_2_, and O_2_ pulse as a function of WR, became abnormal in the period following the AT. Steepening of the HR response as a function of V̇O_2_ with a simultaneous flattening of the O_2_ pulse response and a slowing of the V̇O_2_ response as functions of WR were noted at a WR of 78 watts. Figure 1 illustrates normal HR vs. V̇O_2_ and O_2_ pulse vs. WR responses of the control participant (mentioned in Table 1) compared to the abnormal case participant.

**Figure 2:**
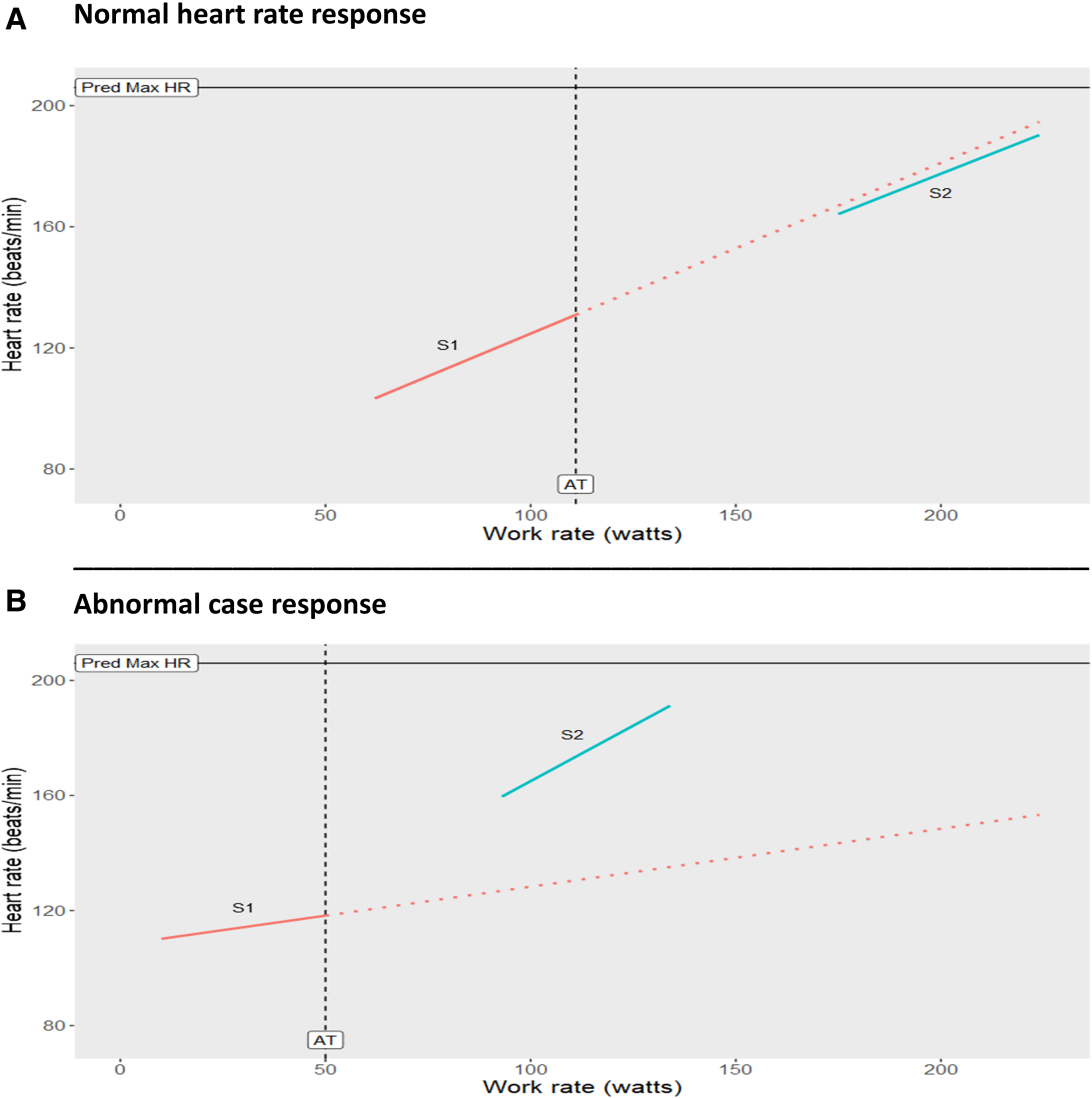
(**A)**: Normal heart rate response seen in a healthy 14-year old male control showing normal cardiac function. Note there is only a slight change in slope (−5%) between the 2 min prior to exercise test termination (S2) and the 2 min prior to AT (S1). (**B**): Abnormal heart rate response from the case participant 14-year old male showing cardiac dysfunction. Note the significant steepening of slope S2 (286%) in late exercise compared to the baseline S1. S1 = slope of line of best fit for heart rate response in the 2 min prior to the exercise test termination. Dashed lines represent trajectory of S1 (baseline slope). AT, anaerobic threshold; Pred Max HR, predicted maximum heart rate.

CPET slopes (ΔV̇O_2_/ΔWR, ΔHR/ΔWR, ΔO_2_ pulse/ΔWR) percent change were calculated by comparing the slope corresponding to the 2 min of exercise prior to test termination (slope 2) to the slope corresponding to the 2 min preceding AT (slope 1, baseline slope). This quantitative analysis showed a 286% steepening in ΔHR/ΔWR in slope 2 compared to slope 1 and a 105% decrease in O_2_ pulse using the same slope comparison process, suggesting an underlying cardiac dysfunction. Table 2 displays these abnormal slope changes compared to the control and other published healthy cohort slope data (1, 2). Figure 2 illustrates the normal HR response of the control compared to the abnormal case participant's HR response.

**Table 2:** CPET slope changes: case participant vs. healthy control and published cohorts.

CPET slopes % change (slope 2 vs. slope 1)	Case participant (adolescent)	Control (healthy adolescent)	Chaudhry et al. (1) (healthy adult cohort)	Van De Sande et al. (2) (healthy adult cohort)
ΔHR/ΔWR	286%	−5%	−6%	−24%
ΔO_2_ pulse/ΔWR	−105%	32%	NA	4%

The case participant has a significant acceleration of HR response and decrease in O_2_ pulse response in late exercise (ΔHR/ΔWR and ΔO_2_ pulse/ΔWR slopes) compared to healthy individuals. CPET, cardiopulmonary exercise testing; ΔHR/ΔWR, change in heart rate relative to the change in work rate slope in the 2 min prior to exercise test termination compared to baseline; ΔO_2_ pulse/ΔWR, change in oxygen pulse relative to the change in work rate slope in the 2 min prior to exercise test termination compared to baseline; NA, not available.

Following the study visit, the abnormal CPET pattern indicative of cardiac dysfunction was identified. The center physician soon informed the parent of the finding and advised them to follow-up with a cardiology appointment. It was then that the parent revealed the case adolescent had a family history that was significant for early-onset coronary artery disease (CAD), with his father having a myocardial infarction before 50 years of age. The participant was seen by a pediatric primary care provider for further evaluation. A resting EKG was performed which showed normal sinus rhythm and no further testing was recommended by the provider. Thus, there was no intervention for the case. Unfortunately, we were not in a position to influence this decision, as it is our opinion that the case adolescent did not get sufficient thorough follow-up testing which should go beyond resting EKG measurements, as further discussed below.

Because of the irregular findings reported here, early serum sample analyses were done for the case and a control participant. It was found that the case participant's serum hs-CRP was elevated at 5.3 mg/L vs. 0.1 mg/L in the control participant (5%–95% interval in children: 0.1–2.8 mg/L) (5) (Table 1).

## Discussion

Previous CPET research has mostly focused on symptomatic individuals and few investigative studies have been published utilizing CPET to assess the cardiovascular and pulmonary function in asymptomatic at-risk individuals (6). However, evidence of the utility of CPET as a unique diagnostic tool is emerging. This case highlights the contrast between a healthy adolescent control at low risk for cardiovascular disease and an adolescent at a significantly increased risk due to family history of paternal early-age CAD and being in the overweight BMI percentile. The abnormal CPET responses indicative of cardiac dysfunction combined with elevated hs-CRP levels add further importance to the need for a more thorough clinical evaluation. Therefore, the evidence highlighted in this case raises awareness to the potential value of using CPET in this at-risk population and the need for further testing and monitoring beyond a resting EKG and other common resting measures (e.g., blood pressure, cholesterol, etc). As such, CPET may contribute to early detection of cardiopulmonary abnormalities, which again would enable early interventions.

During CPET in healthy individuals, there is little change in the trajectory of key parameters in late exercise compared to early exercise when using a continuous linear WR protocol on a cycle ergometer (Figure 1, panels A and B). A normal AT, ΔV̇O_2_/ΔWR, and peak V̇O_2_ (relative to the percent predicted 3, 4) in response to exercise is required for an individual to have appropriate oxygen transport and physiological function. Substantial steepening in the HR slope and significant decrease in the O_2_ pulse slope after the AT in the case participant, reflect a compensatory HR response caused by the inability to augment stroke volume (7) (arteriovenous O_2_ difference normally increases relatively linearly from rest to peak V̇O_2_ (8)) — all while WR and minute ventilation increased linearly. These findings, combined with reduced AT, ΔV̇O_2_/ΔWR, and peak V̇O_2_ are typical physiological markers of inadequate oxygen transport. Since a slowing of ΔV̇O_2_/ΔWR was observed in late exercise, rather than a plateau, it may suggest the presence of a modifiable early-stage disease state. In adults, the significant early plateauing of ΔV̇O_2_/ΔWR and ΔO_2_ pulse/ΔWR is often indicative of exercise-induced myocardial ischemia, a late-stage manifestation of obstructive coronary artery disease (9, 10). Interestingly, a case report by Chaudhry et al. (11) detailed CPET's ability to detect and track the progression and regression of cardiac dysfunction in an asymptomatic young adult with a strong family history of early-age CAD. Therefore, our case findings suggest cardiac dysfunction may be detected by CPET at an even earlier age.

Obesity is characterized with a low-grade systemic inflammation. However, as an adjunct screening tool to lipid screening, the predictive role of hs-CRP in the future occurrence of cardiovascular diseases in at-risk adults has been recognized (12). This is particularly important given that a significant percentage of adults with CAD have normal LDL cholesterol at hospital admission (13). In fact, a recent publication detailing hs-CRP levels in a large multi-cohort of adolescents with varying metabolic phenotypes provides strong evidence that the abnormal case participant CPET responses are grossly different from adolescents who are metabolically unhealthy and obese (14). This suggests that findings in the case adolescent may not simply be attributed to an unhealthy metabolic phenotype or excess weight.

Comparing slope changes of key CPET parameters of the case participant to the slope changes of the control and other published adult slope data (1, 2) make these abnormalities evident (Table 2). Although abnormal, there is no evidence suggesting to terminate a CPET prematurely when the individual is asymptomatic throughout testing. Recently, a study assessing early flattening of O_2_ pulse in asymptomatic middle-aged adults found it was associated with cardiovascular risk factors (15). This data strengthens our findings, which are novel due to the young adolescent age of the case participant. Significant differences in cardiorespiratory fitness have been found in children and adolescents when comparing those with normal weight to those with obesity (16). Usually, these studies focus on peak V̇O_2_ as the primary outcome and do not assess for abnormal patterns that may reveal disease states, as we did in ours. This highlights the role of CPET in early detection of abnormal patterns, which would enable early intervention as long as the importance of the findings are recognized and proper follow-up is given. Further, a plan that incorporates CPET for prognosis of future cardiovascular diseases in at-risk asymptomatic adolescents is again novel, but presents challenges as described below. The current findings highlight the need for more research into abnormal CPET responses and their causes in this population, and identify best management practices.

One of the barriers to identifying a pathology following an abnormal CPET in young persons may be the assumption that subtle abnormalities are not too concerning since adolescents are viewed as being “naturally healthy” despite growing reports of negative health effects of poor diet and physical inactivity at young ages. This age bias may result in little to no follow-up testing after an abnormal CPET, especially in asymptomatic individuals, as in the example of this case. Since CPET research in this population is in its infancy and this testing is largely reserved for clinical populations, there are no evidence-based guidelines available for clinicians.

Likewise, it is known that some abnormalities are only first revealed during exercise, as in the example of this case. For instance, diastolic dysfunction, microvascular disease and endothelial dysfunction can cause abnormal CPET responses similar to this case, but the likelihood of identifying those disease states using the standard follow-up resting tests in an asymptomatic adolescent is low. In adults with suspected CAD, compared to ECG-only cardiac stress testing, CPET had superior sensitivity (88% vs. 48%) and specificity (98% vs. 55%) in detecting and excluding exercise-induced myocardial ischemia when nuclear SPECT imaging and coronary angiography were used to determine true CAD (10). Doppler echocardiogram provides valuable information on left ventricular function at rest, however, in asymptomatic apparently healthy adolescents, heart size and ejection fraction are usually normal. Without the evidence provided by CPET, objective signs of evolving pathologies may be absent. Thus, CPET is probably the most sensitive and comprehensive test available when screening for development of disease in high-risk individuals. An effective diagnostic work flow following an abnormal CPET must be driven by the CPET pathophysiological pattern that points toward the organ of cause. Therefore, work-up beyond the classic resting tests are likely needed. As the use of CPET increases in the clinical setting, providers should receive appropriate training to understand the role of CPET in disease detection and management.

## Management considerations

In adolescents with strong family histories of CAD, initial screening for cardiac dysfunction using CPET with simultaneous measures of hs-CRP has potential to provide valuable information on the cardiovascular status and prognosis for early-age CAD risk. At-risk adolescents with abnormal CPET responses and elevated hs-CRP should receive a structured management process, as it may indicate early asymptomatic cardiac dysfunction in the presence of a proinflammatory state. Inflammation has a well-established connection with atherosclerosis development in adults, further underscoring the importance of addressing these findings in a timely and appropriate manner. When abnormal CPET responses are revealed, peak V̇O_2_ and O_2_ pulse (stroke volume) as percent of predicted values may be used to assess to what extent the dysfunction is affecting health status. In general, peak V̇O_2_ and O_2_ pulse in the range of 70%–84% of predicted is classified as a mild cardiopulmonary impairment. However, if peak V̇O_2_ and O_2_ pulse are moderately to severely affected (<70% of predicted), a referral to a pediatric cardiologist could be considered for further assessment and treatment as needed. Serial CPET testing can be utilized to assess the effectiveness of the intervention with the goal of improving peak V̇O_2_ and O_2_ pulse compared to the initial CPET result. Even if a definitive diagnosis cannot be reached after a thorough clinical evaluation, an abnormal CPET result should not be ignored as it may be an early sign of an evolving disease process. Lifestyle interventions utilizing structured diet and individualized exercise prescriptions to target modifiable cardiovascular risk factors may prove to reduce the inflammatory environment and has the potential to normalize the cardiac dysfunction if the driver of the dysfunction is in a modifiable state. Serial CPET testing may help clinicians determine the optimum interval for further workup. Information gained from CPET may be a valuable tool for preventive medicine and should be made more accessible for asymptomatic adolescents who have an increased risk for cardiovascular disease. Collaborating with clinical exercise physiologists with experience in CPET testing and interpretation can optimize clinical workflow, and also underscores the important role of exercise physiology in healthcare. Currently these results, since from a unique test, cannot be effectively applied in a universal way due to barriers previously mentioned. However, we urge clinicians and policy makers not to underestimate the risk and consequences of early-age CAD. We call for studies aiming for a better understanding of early-age CAD with a particular focus on investigation of effective early detection testing and diagnoses in at-risk adolescents to inform treatment and reduce major adverse cardiac event risk.

## Conclusion

Current resting tests and measures are often ineffective at detecting subtle cardiopulmonary abnormalities and cannot detect exercise-induced abnormalities. A detailed assessment of key CPET slopes may enhance sensitivity in detecting abnormalities in asymptomatic at-risk individuals such as the adolescent highlighted in this case report. Serial CPET measures might be important to perform in adolescents with strong family histories of CAD, especially early-age CAD. Research in this at-risk population should strive to better define the clinical role of CPET in the evaluation of cardiac dysfunction for the purpose of improving preventive healthcare. As the use of CPET grows, so will the need to improve healthcare practices and education to incorporate the pathophysiology that CPET may reveal in asymptomatic adolescents at-risk for cardiovascular events.

## Data Availability

The raw data supporting the conclusions of this article will be made available by the authors, without undue reservation.
